# Time-Cumulative Toxicity of Neonicotinoids: Experimental Evidence and Implications for Environmental Risk Assessments

**DOI:** 10.3390/ijerph17051629

**Published:** 2020-03-03

**Authors:** Francisco Sánchez-Bayo, Henk A. Tennekes

**Affiliations:** 1 School of Life and Environmental Sciences, The University of Sydney, Eveleigh, NSW 2015, Australia; 2 ETS Nederland BV, Frankensteeg 4, 7201KN Zutphen, The Netherlands; info@toxicology.nl

**Keywords:** time-dependent toxicity, bees, non-target arthropods, ecological risks, time-to-event bioassays

## Abstract

Our mechanistic understanding of the toxicity of chemicals that target biochemical and/or physiological pathways, such as pesticides and medical drugs is that they do so by binding to specific molecules. The nature of the latter molecules (e.g., enzymes, receptors, DNA, proteins, etc.) and the strength of the binding to such chemicals elicit a toxic effect in organisms, which magnitude depends on the doses exposed to within a given timeframe. While dose and time of exposure are critical factors determining the toxicity of pesticides, different types of chemicals behave differently. Experimental evidence demonstrates that the toxicity of neonicotinoids increases with exposure time as much as with the dose, and therefore it has been described as time-cumulative toxicity. Examples for aquatic and terrestrial organisms are shown here. This pattern of toxicity, also found among carcinogenic compounds and other toxicants, has been ignored in ecotoxicology and risk assessments for a long time. The implications of the time-cumulative toxicity of neonicotinoids on non-target organisms of aquatic and terrestrial environments are far reaching. Firstly, neonicotinoids are incompatible with integrated pest management (IPM) approaches and secondly regulatory assessments for this class of compounds cannot be based solely on exposure doses but need also to take into consideration the time factor.

## 1. Introduction

Neonicotinoids are the most commonly used insecticides in the world, comprising a third of the world market of insecticides [1]. Since the introduction of the first neonicotinoid, imidacloprid, on the market in 1991 several new compounds with the same mode of action have been developed and commercialised for a wide range of uses: from agricultural pest control in more than 150 different crops and extensive use in animal husbandry (i.e., parasite control on sheep) to parasite control in domestic pets and termite control [2]. 

The key to their success is not only the specificity of action against insects and other arthropods but also their versatility, since neonicotinoids are systemic chemicals that can be applied not only as sprays over the crops but also as soil drenches, soil granules and seed-coatings [3]. In fact, the latter has become the most widely used form of application in agriculture, particularly among broad-acre crops such as cotton, maize, oilseed-rape (canola), sugarcane and sunflower, since the water solubility and chemical properties of neonicotinoids ensure their diffusion within treated plants by xylemic and phloemic transport. In addition, it is mainly for this reason that they also impact the environment of the applied areas and beyond, due to their high mobility in soil and contamination of ground- and surface water [4].

To evaluate the environmental impacts of neonicotinoids requires an understanding of the particular mode of action and toxicity of these chemicals. This constitutes the objective of this paper. Neonicotinoids are agonists of the nicotinic acetylcholine receptors (nAChR) that are present in the post-synaptic membranes of all neuronal cells of animals, vertebrates and invertebrates alike. Neonicotinoids bind specifically to the α4β2 subunit of these receptors, which is the common nicotinic subunit in all insects, whereas it makes up only a small fraction of the vertebrates’ nAChRs [5]. For this reason, neonicotinoids are selective insecticides that display much lower acute toxicity towards vertebrates [6]. 

Neonicotinoid molecules compete with the natural neurotransmitter acetylcholine for the same receptors, but with one important difference: while acetylcholine is immediately released and metabolised by the enzyme acetylcholine esterase after binding, neonicotinoids cannot be metabolised by this enzyme and remain bound to the receptors. Consequently, the activation of the receptors by neonicotinoids is not temporary but rather permanent, as the xenobiotic molecules block irreversibly the nAChRs, thus leading to a lethal hyperactivity of the nerves and muscles of the insect [7]. This mode of action elicits a continuous excitation that results in convulsions, trembling, paralysis, and physiological damage, eventually causing the death of the neurons and muscles. These lethal effects are obviously irreversible and different from the behavioural effects, such as feeding inhibition, also observed with some neonicotinoids [8]. However, the reversibility of some behavioural effects induced by imidacloprid cannot be regarded as sufficient evidence for the reversibility of neuronal injury, as they could be due to adaptive processes in surviving neurons—other neonicotinoids do not induce feeding inhibition.

However, the fact that insects and other animals poisoned with low exposure levels of neonicotinoids do not die immediately but rather after prolonged exposure has been described as ‘delayed toxicity’ [9]—as opposed to the typical acute lethality caused by other toxic chemicals—and suggests that the lethal effect of neonicotinoids occurs after the animal has surpassed a critical level of neuronal deaths [10,11]. Such lethal neuronal threshold may be reached in a few hours when animals are exposed to high levels of the chemical or may take days when the exposure concentrations are low—hence the appropriateness of the term ‘delayed’. Consequently, the toxicity of neonicotinoids should not be understood only in terms of acute lethality but must be considered within a chronic framework, as the time to cause a lethal effect can be stretched several days. This pattern, derived from the particular mode of action of these chemicals, is referred to as time-cumulative or time-reinforced toxicity [12].

This pattern of toxicity may be regarded as unusual for insecticides, which are typically chemicals with high acute toxicity, but is nevertheless quite common among other substances. Its theoretical basis was proposed by Druckrey and Küpfmüller in the 1940s [13], and has been confirmed experimentally for several carcinogenic chemicals [14,15], rodenticides [16], the succinate dehydrogenase inhibiting (SDHI)-fungicide boscalid [17], and elementary toxicants such as mercury [18]. We have previously described in detail this pattern of toxicity and indicated a simple way to identify it [10]. In this paper, we only provide a brief description of the mechanisms involved, while focusing mainly on the experimental evidence that demonstrates that neonicotinoids follow such a pattern, with the consequences this entails for a proper risk assessment of these chemicals [19].

## 2. Time-Dependent Toxicity

The classical foundation of toxicology that the ‘dose makes the poison’ (*Dosis facit venenum*, Paracelsus) acquired a second dimension when Fritz Haber established that time of exposure determines the effective toxic dose. Haber’s rule, which establishes that the dose that causes a toxic effect (E) is the product of the applied dose (d) and the time of exposure (t): E = d × t, set the basis for a better understanding of the toxicity of chemicals, and was later analysed and explained mathematically by Druckrey and Küpfmüller [13]. The theoretical work of the latter authors gave a solid foundation to the toxicological science, enabling the prediction of effects with prolonged exposure to chemicals, predictions that were later confirmed empirically.

The underlining assumptions of the Druckrey-Küpfmüller theorem apply to chemicals with specific mode of action such as pesticides targeting enzymes and/or receptors within particular metabolic pathways [20]. As a description of their theorem is outside the scope of this paper, the reader is referred to Tennekes and Sánchez-Bayo 2013 [10] for a complete explanation. Here we summarise the main points that need to be considered for understanding the time-dependent toxicity of neonicotinoids and other toxic chemicals. As neonicotinoids are known to interact with nACh receptors, we use the term receptor, but this description may actually apply to any target molecule where interaction may result in a toxic effect, such as enzymes, DNA, structural proteins, etc. For ease of explanation, we use concentrations instead of doses applied.

A toxicant molecule interacts with a receptor in a bimolecular reaction and the relative concentration of bound receptors (C_R_/R_0_) is assumed to determine the effect. Experimentally, the measured effect is typically used as a proxy of the extent of the interactions, since the proportion of bound receptors cannot be measured directly [21]. 

The kinetics of receptor binding are governed by two rate constants: the time constant for association T_A_ and the time constant for dissociation T_R_. In a steady-state equilibrium, it can be demonstrated that the relative concentration of bound receptors (C_R_/R_0_) is

(1) $$\frac{C_{R}}{R_{0}}=\;\frac{\left[C/R_{0}\right]\cdot \left[T_{R}/T_{A}\right]}{1+\left[C/R_{0}\right]\cdot \left[T_{R}/T_{A}\right]}$$

where C is the concentration at the site of action, which results from the equilibrium between the external and internal concentrations of the toxicant. Please note that in laboratory toxicity tests, the external concentration (c) is assumed to be proportional to the concentration at the site of action C. Equation (1) indicates that the relationship between the relative concentration of bound receptors (C_R_/R_0_) and the relative toxicant concentration (C/R_0_) follows a hyperbolic curve, with saturation of the receptors at very high concentrations (see Figure 2 in [10]). The ratio T_R_/T_A_ determines the strength of the binding and, therefore, of the toxic effect: the higher the ratio, the higher the toxicity. Consequently, substances with large T_R_/T_A_ ratios require small relative toxicant concentrations (C/R_0_) to produce an effect, whereas those with ratios below 1 require higher relative toxicant concentrations. 

Furthermore, the speed in producing an effect depends on the values of the time constants. Low values of T_R_ and T_A_ indicate that both association and dissociation are fast processes, with the toxic effect appearing quickly and the recovery being also quick; the maximum effect will occur when the concentration at the site of action C is highest, and effects will be dependent almost exclusively on concentration. However, a high value of the dissociation constant T_R_ indicates the interaction is slowly reversible, implying that the binding will last a long time. The maximum effect in this case will be delayed as the equilibrium between C and receptor binding will take some time. A high value of the dissociation constant T_R_ will therefore make toxicity time-dependent. A period of latency may occur in which no toxic effects are observed until the proportion of bound receptors C_R_ reaches a certain threshold. 

Of particular interest is the case of toxicants that show irreversible binding to the target receptor, as it is found with neonicotinoids [7], organophosphorus compounds [22,23], synthetic pyrethroids [24], rodenticides inhibitors of vitamin K [16], herbicides inhibitors of photosynthetic pathways or other enzymes [25], and various fungicides and biocides [20]. In such cases, the dissociation constant T_R_ approaches infinity. If the concentration at the site of action C is constant, as it happens whenever exposure concentration c is kept constant throughout a laboratory study, and assuming the bulk of receptors in the organism are not bound to the toxicant (i.e., when C_R_ << R_0_, first-order kinetics) then Equation (1) reduces to

(2) $$\frac{C_{R}}{R_{0}}=KCt$$

here K is the velocity of the process and has the dimension of reciprocal of time. Assuming also there is proportionality between the concentration at the site of action C and the effect E, then

(3) E=KCt

and the toxicant will follow Haber’s rule. In this case, the velocity of the effect (E/t) would be linearly related to the concentration at the site of action C, while the concentration of bound receptors C_R_ would be proportional to the integral of C over time:

(4) $$C_{R}\sim \int^{\;}C\;dt$$

If the subsequent effect happens to be irreversible as well, the effect E would be proportional to the integral of the concentration of bound receptors C_R_ over time:

(5) $$E\sim \int^{\;}C_{R}\;dt$$

When a toxicant has irreversible binding to a receptor and the effect produced is also irreversible (e.g., death), the effect E would be proportional to the double integral of the concentration at the site of action C over time, as the combination of Equations (4) and (5) shows:

(6) $$E\sim \int^{\;}\int^{\;}C\;dt$$

The implication of Equation (6) is that exposure time will enhance the effect E for a given concentration C. Integration of Equation (6) results in E = C × t^2^. 

Haber demonstrated empirically the validity of Equation (3) using nerve gas in experimental animals, and Druckrey and colleagues demonstrated also empirically that Equation (6) holds true in the case of the carcinogenic substances such as 4-dimethylaminostilbene (4-DAST), diethylnitrosamine (DENA) and ethyl-nitrosurea (ENU) [14,15,26]. As the effect of time was more than quadratic in some of their experimental studies, the general form of the Druckrey-Küpfmüller equation is *C* × *t^n^* = constant, where the exponent n can be regarded as an exposure time reinforcement factor. 

The value of the exponent n can be used, therefore, to determine whether a toxicant shows effects reinforced by time (n > 1) or follows Haber’s rule (n = 1). Effects reinforced by time are also called time-cumulative effects, as a given dose will produce effects that build up over time of exposure [10]. We have identified cases of time-dependent toxicity where the value of n is well below 1. Time-dependent effects generally occur when dissociation of bound receptors is a slow process. In the cases where effects are reinforced by time or follow Haber’s rule they are explained by irreversible receptor binding, but in the cases of time-dependent toxicity with n < 1 receptor binding must be assumed to be reversible albeit a slow process. In such a situation high exposure levels are probably more effective than low exposure levels. Table 1 offers a synopsis of the types of dose-response that a toxicant may follow.

## 3. How to Identify Chemicals with Time-Dependent Toxicity

The above theoretical explanation suggests that toxicological data are best understood when the toxic doses of a chemical are referred to specific times of exposure rather than to fixed times, as established in standard toxicity tests (e.g., OECD guidelines). The time-to-effect (TTE) bioassay designed by Newman and McCloskey [27] aims precisely at recording toxic effects over longer periods of time and is suitable for the purpose of analysing time-dependent toxicity of chemicals.

A TTE bioassay requires the same experimental setup as the standard toxicity tests (e.g., range of concentrations, minimum number of individuals and cages, replication) but records the survival or another effect on the organisms over consecutive times (e.g., at 1, 2, 4, 7, 14 and 21 days). Data are tabulated in a matrix for the two variables of interest: chemical concentration (or doses for terrestrial organisms) and time to produce an effect. Consequently, the LC50 or LD50 endpoints can be derived for each one of the times recorded while the time to 50% effect (T50) can be estimated for each of the concentrations used. As with standard toxicity tests, a suitable range of concentrations should be determined prior to conducting a TTE bioassay to ensure that both the toxicity endpoints and T50s can be calculated. The information obtained from the TTE bioassays is superior to that from standard toxicity tests, as it allows identification of time-dependent toxicity patterns in addition to calculating the dose-dependent endpoints. A good example of this procedure can be found in Simon-Delso et al. 2018 [17].

A simple way to scrutinize time-dependent toxicity of a chemical is to look at the total doses that effectively cause an effect within a certain period. When the total doses to cause an effect do not change significantly during the observation period, then the chemical follows Haber’s rule and its toxicity is dependent on both dose and exposure time. However, when the total doses to cause an effect decrease progressively throughout the test period there is proof that the toxicity of the chemical is reinforced by time, i.e., it is time-cumulative. Conversely, when the total doses to cause an effect increase with the exposure time this suggests that although time-dependency may have been observed, the toxicity of low doses is less effective. This could occur when the chemical is metabolised quickly to non-toxic forms by the organism or rapidly eliminated; and the toxicity of the chemical is most effective at high dose. Examples of these three possible cases are presented in Table 2. 

Another way to find out the toxic dose-response pattern of a chemical is to fit a regression line to the experimental endpoints, which are usually estimated as median lethal doses (LD50s) or concentrations (LC50s) against the exposure time (or ED50s and EC50s for non-lethal effects). 

Most authors who carry out toxicity experiments over long periods do not usually estimate such endpoints but instead indicate the T50 values for each concentration C or dose tested. In any case, a linear fit is obtained when using logs for the two variables:

LnT50 = a + b LnC(7)

The result is the same whether using calculated toxicity endpoints or actual tested concentrations, because the T50 for a given concentration indicates the LC50 or EC50 at that precise time [29].

**Figure 1:**
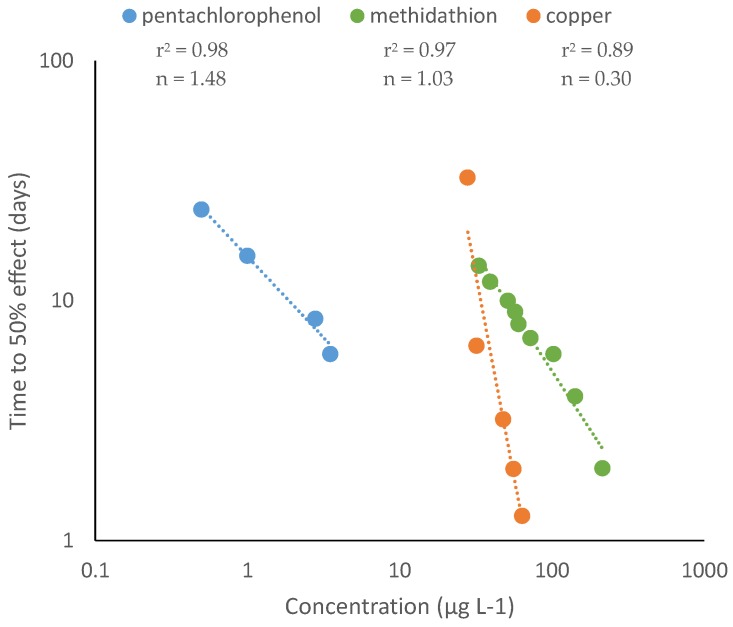
Time-dependent toxicity identified by log-log regression. Time-cumulative toxicity of the biocide pentachlorophenol to the amphipod *Gammarus pulex* (after Ashauer et al. 2007 [30]). Toxicity of the insecticide methidathion on guppies (*Poecilia reticulata*) is time-dependent and follows Haber’s rule (after Legierse et al. 1999 [22]). The toxicity of copper to *Daphnia magna* is mainly dose-dependent (after Hoang et al. 2007 [31]).

Equation (7) allows estimation of the exponent n, which is calculated as the absolute value of the inverse of the slope in the regression: n = 1/|slope|. As described above, values of n = 1 indicate the chemical is time-dependent and follows Haber’s rule. Values of n >1 indicate time-cumulative or reinforced toxicity, whereas values of n <1 indicate the toxicity of the chemical is mainly dose-dependent. Examples of the three types of toxicity patterns determined by log-log regressions are shown in Figure 1.

Given the simplicity with which time-dependency can be scrutinized, the above procedures should be readily incorporated in the standard OECD toxicity tests. One obvious hurdle is that most test guidelines refer to fixed timeframes, so researchers are not required to determine the endpoints at other times than those specified in the guidelines. This shortcoming, which prevents identification of time-dependent toxicity, can be easily overcome by extending the current fixed-time tests so they become variable-time tests (i.e., TTE bioassays). 

## 4. Experimental Evidence for Neonicotinoids

Aware of the delayed mortality and chronic effects of neonicotinoids in organisms, some researchers have conducted laboratory experiments aimed at measuring lethal endpoints for neonicotinoids at various times of exposure. Experimental designs differ markedly among researchers and do not always conform to the TTE bioassay described above, but are often sufficient to estimate toxicity trends with time. Although not exhaustive, a list of cases where neonicotinoids have shown to have time-cumulative lethality on particular organisms is discussed below. The original data reported by the various authors comprise either LC50 values for variable times or T50 values for individual concentrations, which allowed us to perform a regression between both parameters, as in Figure 1.

Terrestrial and aquatic organisms are treated separately because the exposures are different. Terrestrial organisms are fed regularly on food laced with the chemical being tested, so the total dose that produces an effect is calculated multiplying the concentration of the treatment by the amount of food ingested daily and/or for the entire duration of the bioassay. Aquatic organisms are immersed in solutions spiked with known concentrations of the chemical being tested. The actual doses taken up by the organisms are unknown, as they depend on variable uptake through the epidermis, gills and ingested food (chronic experiments require regular feeding). However, as the internal doses are proportional to the external concentrations of chemical in the media, the effects can be related directly to such concentrations.

### 4.1. Aquatic Organisms

Information for 36 tests and four different neonicotinoid compounds was analysed, and the results are shown in Table 3. The data collected refers to eight species of aquatic insects belonging to four different taxa, plus five species of crustaceans from four taxa and one molluscan species. All species tested are freshwater organisms, as the scarce information available on estuarine and marine organisms refers only to acute toxicity and did not allow analysis of time-dependent toxicity [32]. 

In all cases, the species tested showed time-cumulative toxicity, as indicated by values of the exponent n >1. Typical values of n range between 1.2 and 2.5, with only three cases in which n is higher: 2.9 for imidacloprid on *Aedes aegypti* [33], 3.1 for clothianidin on *Chironomus dilutus* [34] and 4.7 for imidacloprid on *Cypridopsis vidua* [29]. The regression coefficients (r^2^) obtained in all cases are >0.80 except for the amphipod *Gammarus pulex* exposed to thiacloprid, which is 0.72. Values of r^2^ = 1.0 are obtained when comparing only two LC50 values because the authors did not report LC50s for intermediate times within the entire exposure period. 

**Figure 2:**
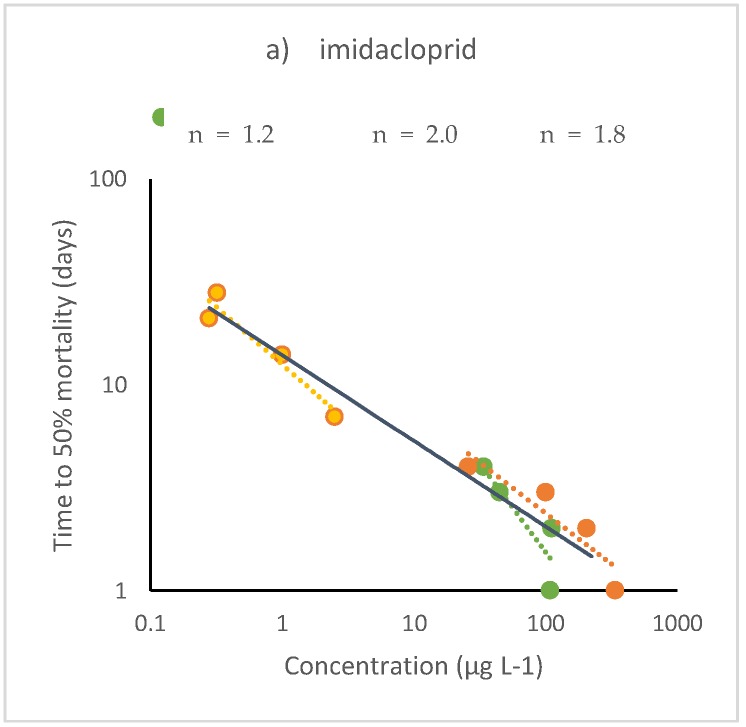

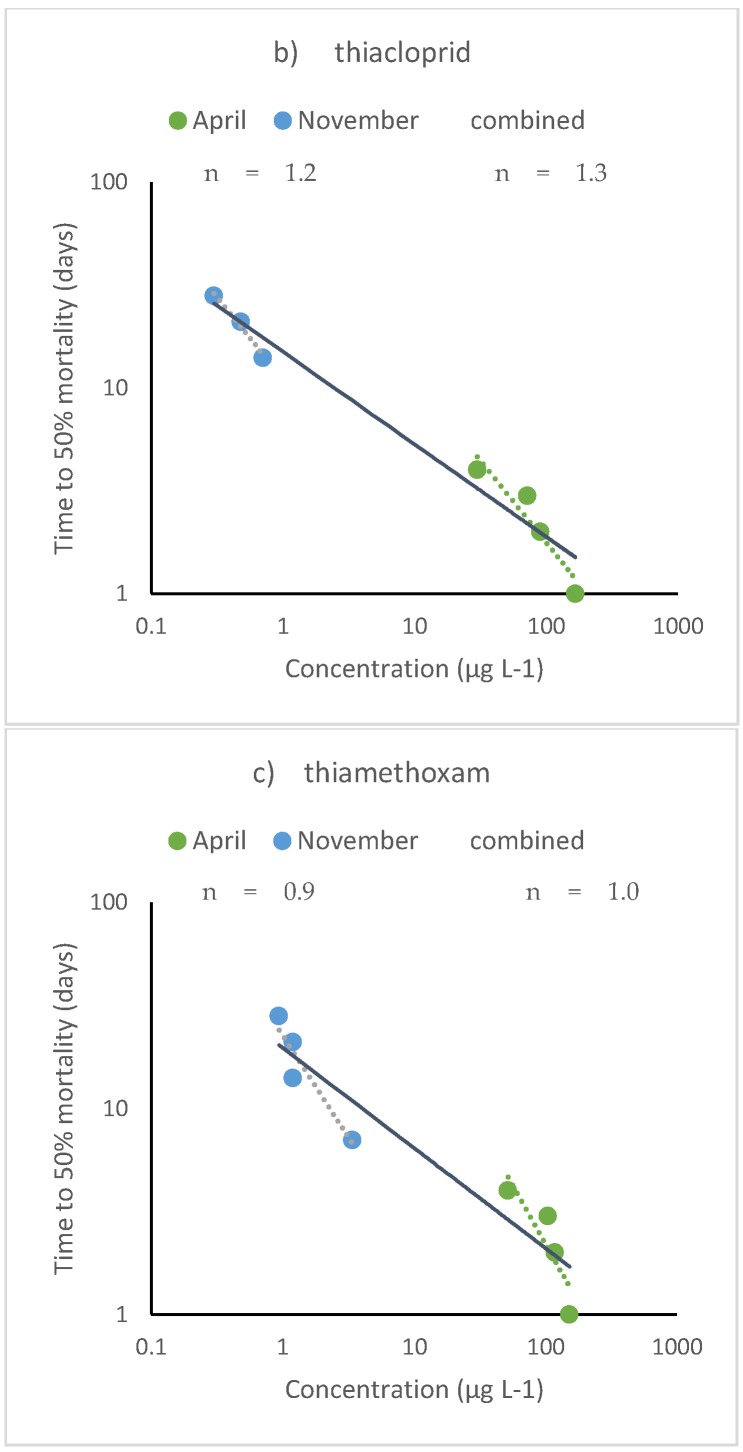
Time-cumulative toxicity of (**a**) imidacloprid, (**b**) thiacloprid and (**c**) thiamethoxam tested on nymphs of the mayfly *Cloeon dipterum* (after van den Brink et al. 2016 [39]). Tests were carried out separately for acute (up to 4 days) and chronic toxicity (up to 28 days), with individual tests showing values of n ≥1 (dotted lines). The combined sets also show values of n >1 (solid lines), confirming the time-dependent toxicity of all three compounds.

It is noted that the decrease in LC50 values (ΔLC50) between the initial and final exposure times varies markedly. Differences are smaller for clothianidin (3 to 9 times) and larger for imidacloprid and thiamethoxam (5 to 28 times), regardless of the length of the exposure period. While the sensitivity among species is obviously different, the largest ΔLC50 values are obtained when comparing acute and chronic LC50s determined in split experiments (Figure 2a–c). For example, van den Brink et al. 2016 carried out sets of acute toxicity tests with nymphs of the mayfly *Cloeon dipterum* using a range of concentrations and determined the LC50s for 1, 2, 3 and 4 days. They also carried out another set of chronic toxicity tests using a lower range of concentrations and determined the LC50s for 7, 14, 21 and 28 days. When comparing the 1-d LC50 values to the 28-d LC50 values, the ΔLC50 are 187–700 for imidacloprid, 190–557 for thiacloprid and 131–163 for thiamethoxam. In the case of imidacloprid, the largest difference corresponds to tests carried out in the spring-summer season (April-August) and the lowest to those performed in autumn (October-December). For thiacloprid and thiamethoxam, the largest value refers to LC50 endpoints and the lowest to EC50 endpoints [39]. Note also that whenever the T50 only was reported, no ΔLC50 could be determined.

It should also be noted that six additional tests carried out by van den Brink et al. 2016 with the mayfly nymphs followed Haber’s rule. Two of them used imidacloprid and the others thiamethoxam; data from another test could not be used (Table S1). The same finding applies to two tests using imidacloprid: one on the amphipod *Gammarus pulex* [38] and another on the midge *Chironomus riparius* [42]. A further test using thiacloprid on the caddisfly *Notidobia ciliaris* suggests the same pattern, as indicated by n = 0.91 despite the poor fit of the model to the data (r^2^ = 0.47) [9].

We emphasize that testing conditions are important for an accurate determination of LC50 or T50 values, which in turn can influence the calculation of n values in the model regression. For example, acute tests (4 days) with thiamethoxam on the amphipod *Gammarus kischineffensis* showed a clear time-cumulative pattern (n = 2.41) due to the excellent fit to the estimated LC50 values (r^2^ = 1.0) [40], whereas the same compound tested on the same species under different conditions showed n = 0.80, which is close to following Haber’s rule [43]. The latter test, however, showed poorer goodness of fit to the data (r^2^ = 0.87) than the former one.

### 4.2. Terrestrial Organisms

Information for 27 tests and four different neonicotinoid compounds was analysed, and the results are shown in Table 4. The data collected refers to 14 species of insects belonging to seven different taxonomic orders; among them is the brown planthopper (*Nilaparvata lugens*), a pest of rice crops and *Frankiniella occidentalis*, a pest of many flowers. In addition to the above, two tests using clothianidin showed that this compound followed Haber’s rule when tested on honeybees (*Apis mellifera*) [44,45], whereas the same compound tested on bumblebees (*Bombus terrestris*) and mason bees (*Osmia bicornis*) appeared to show mainly dose-dependent toxicity [45]. One additional data set included acetamiprid, but this compound did not show time-dependent toxicity when tested on honeybees [46] (Table S2), perhaps because it is quickly metabolised [47].

**Figure 3:**
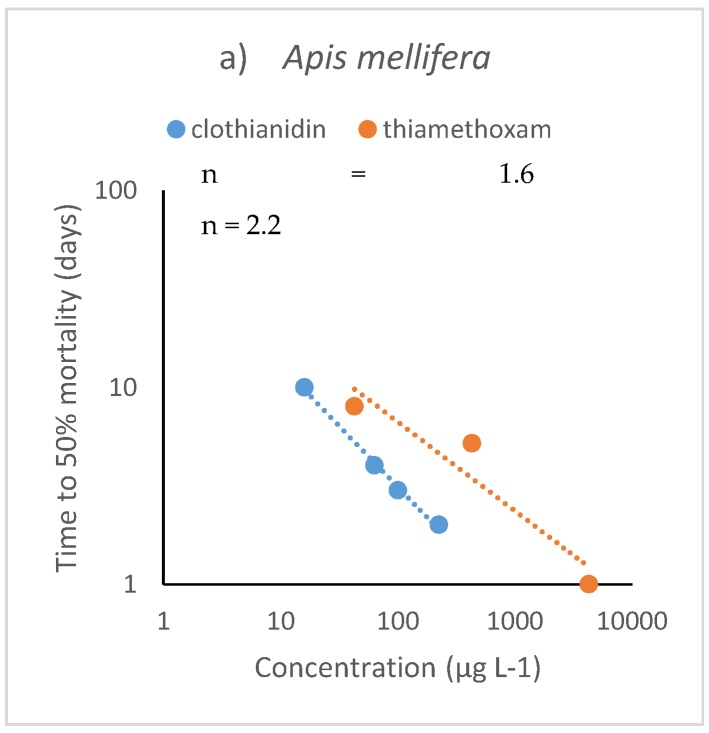

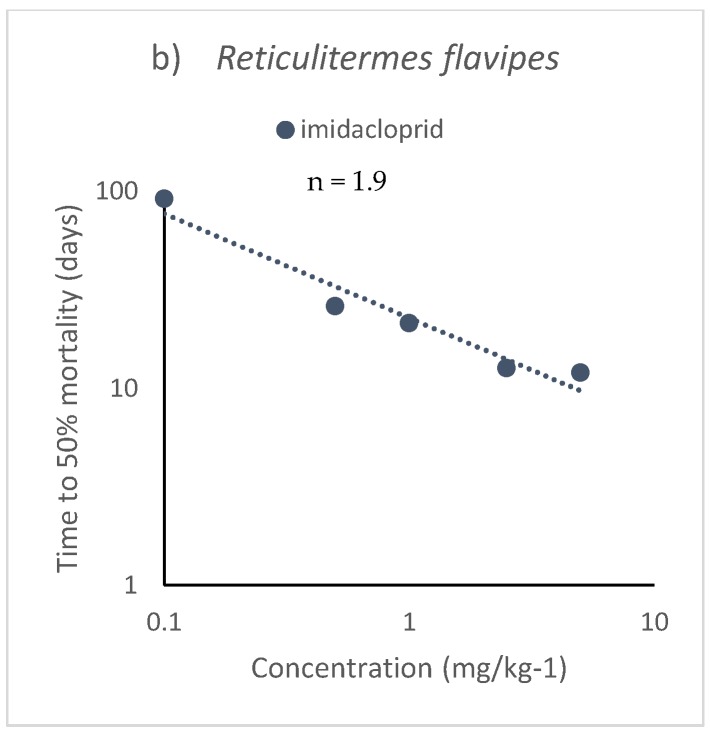
Examples of time-cumulative toxicity in terrestrial insects for (**a**) clothianidin and thiamethoxam on honey bees (*Apis mellifera*) [49,61]; (**b**) imidacloprid on termites *Reticulitermes flavipes* (sandy soil) [59].

Typical values of n obtained from tests with terrestrial insects range between 1.2 and 2.7, similar to that with aquatic organisms. However, five tests showed higher values of the n exponent: clothianidin on the predatory bug *Cyrtorhinus lividipennis* and the brown hopper pest species indicate values of n = 3.7 and 4.5, respectively [48]; imidacloprid on the ant *Linepithema humile* (n = 3.5) [58], on the termite *Reticulitermes flavipes* (n = 4.0) [59] and on the honeybee (n = 5.8) [54]. The latter value of n is unusually high, suggesting that the bees tested may have died by an interaction of the insecticide with other factors such as pathogenic infections or others. Nevertheless, given the good fit of the model to the dataset (r^2^ = 0.85) this result cannot be excluded. Examples of time-cumulative toxicity for three chemicals are shown in Figure 3a,b.

The decrease in LC50 values (ΔLC50) between the initial and final exposure times varies in the range 3 to 52 for all compounds, with notable exceptions for imidacloprid tested on female fruit flies (*Drosophila melanogaster*) and termites, which showed differences of 172 times over 8 days and 1167–3126 times over 21 days, respectively [51,59]. The result for imidacloprid on the fruit flies contrasts with previous tests that showed no time-dependent toxicity of this compound to the same species and equal 8-day testing period [62]. 

We observe that the length of the testing period does not prevent estimation of the n values provided that the calculated LC50 or T50 values are accurate and show small variance. Conversely, a high variability in the dataset may result in poor fitting of the endpoint values to the regression model and therefore lead to unreliable conclusions. Two of the tests shown in Table 4 fall in the latter category: thiacloprid tested on honeybees [46] showed a time-cumulative pattern (n = 2.1) but poor goodness of fit (r^2^ = 0.44), and imidacloprid tested on adults of the parasitoid *Aphidius colemani* [52] showed also a time-cumulative pattern (n = 2.3) despite the poor fit (r^2^ = 0.59). In both cases the patterns of toxicity may be regarded as unreliable, even if they are time-dependent.

## 5. Implications for Risk Assessment of Neonicotinoids

Research on the influence of time on the toxicity of pesticides spans several decades [28,30,63], and yet most ecotoxicologists do not consider the time factor in their routine toxicity tests. The fact that OECD toxicity test guidelines stipulate fixed times of exposure for both acute and chronic tests is a major hurdle, as chemical regulators only take into consideration toxicity data that conform with such tests. As a result of having insufficient toxicological data, environmental risk assessments are often unable to evaluate the real impacts that chemicals have on organisms [64]. This has become apparent in the case of neonicotinoid compounds, for which the current evidence demonstrates their actual environmental impacts on both aquatic and terrestrial ecosystems are larger than previously estimated [65,66].

When neonicotinoids were first approved as agricultural insecticides, the only toxicity data available were acute endpoints for a limited number of surrogate terrestrial and aquatic species, as required by the regulators. It was later found that the species indicative of risks to the aquatic environment (i.e., *Daphnia* sp.) was unusually tolerant to this class of chemicals [67,68], thus making the assessment of impacts on aquatic organisms completely misleading and not representative of the risks posed to the majority of aquatic invertebrates [69]. Further research on aquatic invertebrates revealed a delayed effect on mortality, especially among aquatic insect species [9] that could not be detected in standard acute tests. In addition to the lethal effects, a plethora of negative sublethal effects on bees as well as in vertebrates [70,71] demonstrate that risk assessments for neonicotinoids have been inadequate to protect the environment.

However, the main deficiency in the risk assessments of neonicotinoids was and still is the consideration of acute endpoint values while ignoring delayed and chronic effects. This should not surprise us, as the time-cumulative toxicity of these compounds was unknown and could not be imagined. This unfortunate situation has now changed. As demonstrated by the evidence shown in Table 3 and Table 4, the toxicity of neonicotinoids is reinforced by time and fit the pattern of toxicity explained by Druckrey-Küpfmüller [19]. This feature explains their slowness in producing toxic effects, as they are delayed, with their acute toxicity endpoints being relatively high in comparison to those of organophosphorus and pyrethroid compounds. It also explains the discrepancy in toxicity endpoints for bees reported in the literature (up to 33-fold), as different authors used different exposure times to estimate them [72]. Thus, values of lethal endpoints decrease markedly between short and long exposures [38], a fact than many researchers could not explain nor understand properly. 

The main implication is that the risks to pollinators and other beneficial non-target arthropods such as predatory insects and parasitic wasps have been underestimated. Constant exposure to neonicotinoid residues in pollen and nectar leads to the eventual death of the forager bees, moths, butterflies and polyphagous parasitoids [73] even if their losses cannot be adequately monitored because the dead insects cannot be found [74]. Most importantly, the toxicity to parasitoids is usually higher than that to the pest species they control [48], which inevitably results in the loss of the natural pest control in agroecosystems. For this reason, neonicotinoids are not compatible with integrated pest management (IPM) approaches in agriculture [75,76], despite claims to the contrary made in studies that only looked at the acute toxicity in standard glass plate tests [77,78]. If we are to protect the biodiversity of beneficial arthropods, which are more efficient in controlling pests than chemicals, neonicotinoids have no place in sustainable agriculture [79], as they undermine the natural pest control systems. 

In aquatic ecosystems, neonicotinoids eliminate the nymphs and other insect larvae that are inevitably exposed to low albeit constant concentrations of these compounds. Previous risk assessments based on acute toxicity endpoints could not find significant risks [80,81] because they ignored the subtle and delayed mortality that takes place as a result of the time-cumulative pattern of toxicity of neonicotinoids. No matter how low their waterborne residues maybe, sensitive species such as caddisflies, mayflies, stoneflies, craneflies and others experience cumulative death tolls that make the recovery of their populations unlikely if not impossible (unless there is re-colonisation from outside sources). Not only insects, but planktonic and benthic crustaceans undergo the same fate and are decimated [82], to the extent that fish populations starve and collapse in turn [83]. As a result, the diverse arthropod communities of freshwater ecosystems collapse, impairing the recycling of nutrients and causing starvation among many insectivorous birds, reptiles and amphibians that depend on them [65]. 

Time-cumulative toxicity of neonicotinoids is repeatedly found among aquatic and terrestrial species of invertebrates alike [84], perhaps because the most common subtype of nAChRs in these organisms is α4β2, which shows the strongest binding affinity towards neonicotinoids [5]. As with vertebrates, it is conceivable that in the least sensitive organisms, such as waterfleas, the nAChR subunits may be different and have little affinity for these chemicals, since metabolism of neonicotinoids appears to be mediated by mono-oxygenase enzymes of the P450 family in all cases [5].

To protect the environment from further chemical impacts, a new framework for risk assessment of chemicals should consider the time-dependent toxicity in the first tier of the assessment [85]. This is particularly urgent for neonicotinoids and other pesticides with irreversible binding to specific receptors, because they are the most likely candidates to undergo toxic effects that are enhanced by time of exposure. 

As neonicotinoids have replaced older pesticide chemistries in most countries, the actual toxic loads of insecticides per area have dramatically increased in recent years [86,87]. It is not surprising, therefore, that current declines of biodiversity have been linked to pesticides in both terrestrial [88] and aquatic environments [89,90,91], among which neonicotinoids are prevalent. Thus, the decline of dragonfly populations in Japan, particularly the most common species of rice paddies, akiakane (*Sympetrum frequens*), is attributed to the use of imidacloprid and fipronil over the past 25 years [92]. Also in Japan, the collapse of fisheries in lake Shinji has being linked to the indirect impact of neonicotinoids used in nearby rice farms [83]. In the Netherlands, the decline of macro-invertebrates in surface waters correlates with residues of imidacloprid among other chemicals [82], and imidacloprid is the main factor behind the decline of insectivorous and granivores songbirds in that country [93]. 

The above are examples adding to the mounting evidence that current risk assessments for registration of chemicals in OECD countries are inadequate to protect biodiversity and ecosystems and need a revamp [94,95,96]. To provide more realistic risk assessments of agricultural and other chemicals, toxicological bioassays designed for detecting the time-cumulative toxicity of substances should be made mandatory [17,85]. Only then we would be able to distinguish chemicals that have time-cumulative and other time-dependent toxicity from those that are mainly dose-dependent.

## Figures and Tables

**Table 1:** Dose-response characteristics according to Druckrey-Küpfmüller.

Bound Receptors in Relation to Toxicant Concentration	Receptor Binding	Effect	Effect in Relation to Bound Receptors	Effect in Relation to Toxicant Concentration	Characteristics *	Value of Exponent n
*C_R_* *~ C*	Reversible *T_R_* → 0	Reversible	*E ~ C_R_*	*E ~ C*	Dose-dependent	n < 1
		Irreversible	$E\sim \int^{\;}C_{R}\;dt$	$E\sim \int^{\;}C\;dt$	Haber’s rule *C·t* = constant	n = 1
$C_{R}\sim \int^{\;}C\;dt$	Irreversible *T_R_* → ∞	Reversible	*E ~ C_R_*			
		reversible	$E\sim \int^{\;}C_{R}\;dt$	$E\sim \int^{\;}\int^{\;}C\;dt$	Time-reinforced *C·t^n^* = constant	n > 1

C_R_ = bound receptors; C = toxicant concentration; T_R_ = time dissociation constant; E = effect; * the assumption made to explain Haber’s rule and time-reinforced toxicity is that C remains constant upon repeated dosing (continuous exposure).

**Table 2:** The three patterns of chemical toxicity with time of exposure as identified by the total dose or C × T50. The carcinogen diethylnitrosamine shows time-cumulative toxicity in rats. The organophosphorus insecticide phosmet shows time-dependent toxicity that complies with Haber’s rule. Toxicity of the reagent cadmium chloride is mainly dose-dependent.

Diethylnitrosamine ^1^			Phosmet ^2^			CdCl_2_^3^		
*Rattus sp.*			*Poecilia reticulata*			*Daphnia magna*		
n = 2.3, r^2^ = 1.0			n = 1.0, r^2^ = 0.96			n = 0.6, r^2^ = 0.98		
Daily dose	T50	Total dose	Concentration (C)	T50	C × T50	Concentration (C)	T50	C × T50
mg·kg^−1^	days	mg·kg^−1^	μM	days	μM	μg·L^−1^	days	μg·L^−1^
9.6	101	963	8	1	8.0	56	2	105
4.8	137	660	5.2	2	10.4	32	6	181
2.4	192	460	3.2	3	9.6	18	11	203
1.2	238	285	2.7	4	10.8	10	38	375
0.6	355	213	2.4	5	12.0	5.6	58	325
0.3	457	137	1.8	6	10.8	3.2	292 *	935
0.15	609	91	1.6	7	11.2			
0.075	840	64	0.93	8	7.4			
			0.8	10	8.0			

C = concentration in water; T50 = median time to effect. * estimated value greater than the life span of the organism. ^1^ Druckrey et al. 1963 [14]; ^2^ Legierse et al. 1999 [22]; ^3^ Kooijman 1981 [28].

**Table 3:** Time-cumulative toxicity of neonicotinoids in aquatic organisms.

Taxa	Species	Chemical	n (1/slope)	Regression Parameters			ΔLC_50_	No. c tested	Exposure Time (days)	Reference
				Intercept	Slope	r^2^				
Diptera	*Aedes aegypti*	CLO	1.70	3.835	−0.588	0.98	7	5	3	Ahmed and Matsumura 2012 [33]
Diptera	*Chironomus dilutus*	CLO	3.11	2.922	−0.322	1.0	9	5	40	Cavallaro et al. 2017 [34]
Ephemeroptera	*Deleatidium sp.*	CLO	1.59	3.515	−0.628	1.0	3	10	28	Macaulay et al. 2019 [35]
Amphipoda	*Hyalella azteca*	IMI	1.58	4.085	−0.634	0.65	8	5	28	Stoughton et a. 2008 [36]
Cladocera	*Daphnia magna*	IMI	2.41	6.540	−0.410	0.89	5	6	10	Sanchez-Bayo 2009 [29]
Cladocera	*Daphnia magna*	IMI	1.91	6.646	−0.523	0.99	21	6	4	Sanchez-Bayo (unpublished)
Cladocera	*Daphnia magna*	IMI	2.56	5.999	−0.390	0.99	na	6	21	Ieromina et al. 2014 [37]
Diptera	*Aedes aegypti*	IMI	2.90	2.771	-0.345	0.99	23	5	3	Ahmed and Matsumura 2012 [33]
Diptera	*Chaoborus obscuripes*	IMI	1.62	4.897	−0.618	1.0	23	5	28	Roessink et al. 2013 [38]
Diptera	*Chironomus dilutus*	IMI	1.21	3.254	−0.825	1.0	na	5	28	Stoughton et al 2008 [36]
Diptera	*Chironomus dilutus*	IMI	1.30	2.962	−0.772	1.0	na	5	40	Cavallaro et al. 2017 [34]
Ephemeroptera	*Cloeon dipterum*	IMI	2.52	2.684	−0.397	1.0	135	5	28	Roessink et al. 2013 [38]
Ephemeroptera	*Cloeon dipterum*	IMI	2.40 *	2.634	−0.416	0.96	700	7	28	Van den Brink et al. 2016 [39]
Ephemeroptera	*Cloeon dipterum*	IMI	2.03	3.137	−0.493	0.84	13	7	4	Van den Brink et al. 2016 [39]
Ephemeroptera	*Cloeon dipterum*	IMI	1.79	2.531	−0.559	0.92	8	7	28	Van den Brink et al. 2016 [39]
Ephemeroptera	*Cloeon dipterum*	IMI	2.11 *	3.037	−0.473	0.80	187	7	28	Van den Brink et al. 2016 [39]
Ephemeroptera	*Cloeon dipterum*	IMI	1.38	3.862	−0.726	0.99	7	7	4	Van den Brink et al. 2016 [39]
Ephemeroptera	*Coenis horaria*	IMI	1.57	2.597	−0.638	1.0	21	5	28	Roessink et al. 2013 [38]
Ephemeroptera	*Deleatidium sp.*	IMI	2.05	2.620	−0.489	0.95	14	10	28	Macaulay et al. 2019 [35]
Isopoda	*Asellus aquaticus*	IMI	1.41	5.466	−0.709	1.0	16	5	28	Roessink et al. 2013 [38]
Megaloptera	*Sialis lutaria*	IMI	2.94	4.515	−0.340	1.0	308	5	28	Roessink et al. 2013 [38]
Ostracoda	*Cypridopsis vidua*	IMI	4.67	5.110	−0.210	0.88	na	6	4	Sanchez-Bayo 2009 [29]
Amphipoda	*Gammarus pulex*	THC	1.30	1.729	−0.767	0.72	na	5	15	Beketov & Liess 2008 [9]
Diptera	*Aedes aegypti*	THC	1.54	4.166	−0.648	1.0	5	5	3	Ahmed and Matsumura 2012 [33]
Ephemeroptera	*Cloeon dipterum*	THC	2.23 *	2.707	−0.449	0.96	557	7	28	Van den Brink et al. 2016 [39]
Ephemeroptera	*Cloeon dipterum*	THC **	1.83 *	2.353	−0.547	0.95	190	7	28	Van den Brink et al. 2016 [39]
Ephemeroptera	*Cloeon dipterum*	THC	1.25	2.398	−0.798	0.98	na	7	28	Van den Brink et al. 2016 [39]
Ephemeroptera	*Cloeon dipterum*	THC	1.25	3.166	−0.801	0.97	6	7	4	Van den Brink et al. 2016 [39]
Ephemeroptera	*Cloeon dipterum*	THC	1.26	4.242	−0.797	0.89	6	7	4	Van den Brink et al. 2016 [39]
Isopoda	*Asellus aquaticus*	THC	1.25	0.932	−0.802	0.94	na	3	19	Beketov & Liess 2008 [9]
Odonata	*Sympetrum striolatum*	THC	1.53	7.430	−0.650	1.0	na	4	11	Beketov & Liess 2008 [9]
Amphipoda	*Gammarus kischineffensis*	TMX	2.41	4.768	−0.416	1.0	28	6	4	Ugurlu et al. 2015 [40]
Diptera	*Chironomus dilutus*	TMX	2.51	3.896	−0.398	1.0	na	5	40	Cavallaro et al. 2017 [34]
Ephemeroptera	*Cloeon dipterum*	TMX	2.05 *	2.980	−0.487	0.91	163	7	28	Van den Brink et al. 2016 [39]
Ephemeroptera	*Cloeon dipterum*	TMX **	1.70 *	2.949	−0.589	0.96	131	7	28	Van den Brink et al. 2016 [39]
Mollusca	*Planorbella pilsbryi*	TMX	1.33	8.521	−0.753	1.0	6	5	28	Prosser et al. 2016 [41]

* Combined data from acute and chronic tests; ** EC50 data; ΔLC_50_ = difference between short- and long-term LC50s; na = not available, as T50 was estimated. CLO = clothianidin; IMI = imidacloprid; THC = thiacloprid; TMX = thiamethoxam.

**Table 4:** Time-cumulative toxicity of neonicotinoids in terrestrial organisms.

Taxa	Species	Comments	Chemical	n (1/slope)	Regression Parameters			ΔLC_50_	No. c tested	Exposure Time (days)	Reference
					Intercept	Slope	R^2^				
Hemiptera	*Cyrtorhinus lividipennis*		CLO	3.74	1.173	−0.268	1.0	13	6	2	Preetha et al. 2010 [48]
Hemiptera	*Nilaparvata lugens*		CLO	4.49	1.885	−0.233	1.0	22	6	2	Preetha et al. 2010 [48]
Hymenoptera	*Apis mellifera*		CLO	1.19	2.538	−0.841	0.94	11	6	3	Laurino et al. 2011 [46]
Hymenoptera	*Apis mellifera*		CLO	1.62	3.980	−0.617	1.0	14	8	10	Alkassab & Kirchner 2016 [49]
Coleoptera	*Strategus aloeus*	Adults	IMI	2.29	2.073	−0.437	1.0	Na	7	3	Martinez et al. 2014 [50]
Diptera	*Drosophila melanogaster*	Males	IMI	1.42	8.654	−0.703	1.0	29	10	8	Charpentier et al. 2014 [51]
Diptera	*Drosophila melanogaster*	Females	IMI	2.18	5.957	−0.460	1.0	172	10	8	Charpentier et al. 2014 [51]
Diptera	*Drosophila melanogaster*	Larvae	IMI	1.67	6.052	−0.598	1.0	52	10	8	Charpentier et al. 2014 [51]
Hemiptera	*Cyrtorhinus lividipennis*		IMI	1.50	4.811	−0.665	1.0	3	6	2	Preetha et al. 2010 [48]
Hymenoptera	*Aphidius colemani*	Adults	IMI	2.29	3.540	−0.437	0.59	na	6	8	D’Avila et al. 2018 [52]
Hymenoptera	*Apis florea*		IMI	2.74	1.177	−0.365	0.98	na	5	2	Husain et al. 2014 [53]
Hymenoptera	*Apis dorsata*		IMI	2.60	1.454	−0.384	0.99	na	5	2	Husain et al. 2014 [53]
Hymenoptera	*Apis mellifera*		IMI	2.41	1.190	−0.416	0.91	na	5	2	Husain et al. 2014 [53]
Hymenoptera	*Apis mellifera*		IMI	5.83	5.190	−0.170	0.85	na	5	10	Suchail et al. 2001 [54]
Hymenoptera	*Apis mellifera*		IMI	2.67	4.836	−0.375	0.94	46	5	10	DEFRA 2007 [55]
Hymenoptera	*Bracon hebetor*	Adults	IMI	1.80	2.387	−0.554	1.0	3	3	2	Preetha et al. 2010 [56]
Hymenoptera	*Chelonus blackburnii*	Adults	IMI	1.51	5.377	−0.662	0.99	7	3	1	Preetha et al. 2010 [56]
Hymenoptera	*Haeckeliania sperata*		IMI	1.52	−1.039	−0.656	0.92	na	5	2	Carrillo et al. 2009 [57]
Hymenoptera	*Linepithema humile*		IMI	3.47	0.476	−0.288	0.69	na	4	14	Rust et al. 2004 [58]
Isoptera	*Reticulitermes flavipes*	Sand	IMI	1.89	3.125	−0.528	0.95	1167	5	21	Ramakrishnan et al. 2000 [59]
Isoptera	*Reticulitermes flavipes*	Sandy loam	IMI	2.65	3.773	−0.378	0.89	14	7	21	Ramakrishnan et al. 2000 [59]
Isoptera	*Reticulitermes flavipes*	Silty clay loam	IMI	4.00	3.247	−0.250	0.83	3126	7	21	Ramakrishnan et al. 2000 [59]
Thysanoptera	*Frankiniella occidentalis*	Larvae	IMI	1.97	0.495	−0.508	0.92	na	5	8	Niassy et al. 2012 [60]
Hymenoptera	*Apis mellifera*		THC	2.10	1.838	−0.477	0.44	23	3	3	Laurino et al. 2011 [46]
Hymenoptera	*Apis mellifera*		TMX	2.21	4.040	−0.452	0.95	na	3	18	Oliveira et al. 2014 [61]
Hymenoptera	*Linepithema humile*		TMX	1.55	−5.538	−0.643	0.73	na	4	14	Rust et al. 2004 [58]
Thysanoptera	*Frankiniella occidentalis*	Larvae	TMX	1.55	−0.075	−0.645	0.98	na	5	8	Niassy et al. 2012 [60]

ΔLC_50_ = difference between short- and long-term LC50s; na = not available, as T50 were estimated instead. CLO = clothianidin; IMI = imidacloprid; THC = thiacloprid; TMX = thiamethoxam.

## Supplementary Materials

The following are available online at https://www.mdpi.com/1660-4601/17/5/1629/s1, Table S1: Other time-dependent toxicity of neonicotinoids in aquatic organisms, Table S2: Other time-dependent toxicity of neonicotinoids in terrestrial organisms.
