# Supplementary material for: Time-Cumulative Toxicity of Neonicotinoids: Experimental Evidence and Implications for Environmental Risk Assessments
Source: Int J Environ Res Public Health. 2020 Mar 3;17(5):1629. doi: 10.3390/ijerph17051629 (PMC7084546; doi:10.3390/ijerph17051629)
Supplement: Supplementary file 1 [file ijerph-17-01629-s001.pdf]

Table S1. Other time-dependent toxicity of neonicotinoids in aquatic organisms. Bold numbers indicate that the chemical follows Haber's rule.

Taxa	Species	Chemical	n (1/slope)	Regression Parameters			ΔLC_{50}	No. c Tested	Exposure Time (days)	Reference
				Intercept	Slope	R^2				
Diptera	*Aedes aegypti*	ACM	**1.09**	6.037	−0.914	0.99	3	5	3	Ahmed and Matsumura 2012
Diptera	*Aedes aegypti*	DIN	**1.12**	4.994	−0.889	1.0	3	5	3	Ahmed and Matsumura 2012
Amphipoda	*Gammarus pulex*	IMI	**1.05**	6.671	−0.948	1.0	8	5	28	Roessink et al. 2013
Coleoptera	*Hippodamia convergens*	IMI	**0.97**	0.289	−1.035	0.95	na	7	3	Kaakeh et al. 1996
Diptera	*Chironomus riparius*	IMI	**1.13**	3.051	−0.884	1.0	14	8	10	Chandran et al. 2018
Ephemeroptera	*Cloeon dipterum*	IMI	**1.16**	4.393	−0.860	0.76	3	7	4	Van den Brink et al. 2016
Ephemeroptera	*Cloeon dipterum*	IMI	**1.01**	5.539	−0.991	0.25	2	7	4	Van den Brink et al. 2016
Ephemeroptera	*Cloeon dipterum*	IMI	6.45 *	2.861	−0.155	0.11	2	7	28	Van den Brink et al. 2016
Hemiptera	*Plea minutissima*	IMI	0.69	6.642	−1.450	1.0	4	5	28	Roessink et al. 2013
Cladocera	*Daphnia magna*	THC	0.32	6.777	−3.114	0.77	na	3	29	Beketov & Liess 2008
Diptera	*Culex pipiens*	THC	0.43	5.389	−2.327	0.86	na	3	14	Beketov & Liess 2008
Diptera	*Simulium latigolium*	THC	0.63	4.650	−1.595	0.74	na	4	11	Beketov & Liess 2008
Trichoptera	*Notidobia ciliaris*	THC	**0.91**	3.980	−1.101	0.47	na	5	15	Beketov & Liess 2008
Ephemeroptera	*Cloeon dipterum*	THC **	0.81	1.733	−1.231	0.99	3	7	28	Van den Brink et al. 2016
Amphipoda	*Gammarus kischineffensis*	TMX	0.80	12.939	−1.254	0.87	3	10	4	Demirci et al. 2018
Diptera	*Aedes aegypti*	TMX	0.68	7.769	−1.472	0.68	3	5	3	Ahmed and Matsumura 2012
Ephemeroptera	*Cloeon dipterum*	TMX	**0.88**	6.035	−1.139	0.76	3	7	4	Van den Brink et al. 2016
Ephemeroptera	*Cloeon dipterum*	TMX	**1.01**	3.117	−0.986	0.88	4	7	28	Van den Brink et al. 2016
Ephemeroptera	*Cloeon dipterum*	TMX **	**1.08**	4.276	−0.927	0.95	5	7	4	Van den Brink et al. 2016
Ephemeroptera	*Cloeon dipterum*	TMX **	**1.06**	2.975	−0.946	0.95	4	7	28	Van den Brink et al. 2016

* unreliable value due to por fit ($r^2 = 0.11$); ** EC50 data; CLO = clothianidin; IMI = imidacloprid; THC = thiacloprid; TMX = thiamethoxam.

Table S2. Other time-dependent toxicity of neonicotinoids in terrestrial organisms. Bold numbers indicate that the chemical follows Haber's rule.

Taxa	Species	Comments	Chemical	n (1/slope)	Regression Parameters			ΔLC_{50}	No. c tested	Exposure Time (days)	Reference
					Intercept	Slope	R^2				
Hymenoptera	*Apis mellifera*		ACM	0.48	24.942	−2.068	1.0	na	2	3	Laurino et al. 2011
Hymenoptera	*Apis mellifera*		CLO	**1.08**	5.402	−0.926	1.0	6	6	10	Hesketh et al. 2016
Hymenoptera	*Apis mellifera*		CLO	**1.14**	4.811	−0.875	0.99	6	3	10	Heard et al. 2017
Hymenoptera	*Bombus terrestris*		CLO	0.53	7.615	−1.923	1.0	2	3	10	Heard et al. 2017
Hymenoptera	*Osmia bicornis*		CLO	0.27	14.496	−3.711	0.82	1.4	3	10	Heard et al. 2017
Diptera	*Drosophila melanogaster*	Adults	IMI	0.36	28.849	−2.773	1.0	2	5	8	Frantzios et al 2014
Diptera	*Drosophila melanogaster*	Larvae	IMI	0.44	22.082	−2.266	1.0	3	5	8	Frantzios et al 2014
Hemiptera	*Nilaparvata lugens*		IMI	0.33	31.051	−3.015	1.0	1	6	2	Preetha et al. 2010b
Isoptera	*Reticulitermes flavipes*	Loam	IMI	**1.15**	5.947	−0.866	0.85	na	7	21	Ramakrishnan et al. 2000
Hymenoptera	*Apis mellifera*		TMX	**0.95**	4.561	−1.051	0.85	13	7	3	Laurino et al. 2011

CLO = clothianidin; IMI = imidacloprid; THC = thiacloprid; TMX = thiamethoxam.
